# Rare Case Report of Closed Traumatic Dislocation of Second to Fifth Metatarsophalangeal Joints

**DOI:** 10.7759/cureus.11745

**Published:** 2020-11-28

**Authors:** Santhosh Raj, Suresh Subramani, Suraj J Babar, Muthukumar S Balaji, Vijay Anand

**Affiliations:** 1 Department of Orthopaedics, SRM Medical College Hospital and Research Centre, Chennai, IND

**Keywords:** foot and ankle, lesser mtp joints dislocation, irreducible closed reduction, open reduction, dorsal approach, no internal fixation

## Abstract

Closed traumatic dislocation of multiple metatarsophalangeal joints is a rare injury. Until now only one case of simultaneous dislocation of all five metatarsophalangeal joints has been reported in peer-reviewed studies. The complex anatomy of the metatarsophalangeal joints prevents the relocation of the joints in a closed manner in maximum cases. We are reporting a case of dorsal dislocation of the second to fifth metatarsophalangeal joints in the left foot after road traffic accident. Bony prominence over the plantar aspect and increased web space between toes on presentation, then incongruity of metatarsophalangeal joints has to be thoroughly checked on radiograph. Since closed reduction attempts failed open reduction was done through dorsal approach using two incisions. Button holing of the capsule with interposition of capsule and plantar plate was noted. Dorsal approach avoids damage to the plantar plate and surrounding soft tissues.

## Introduction

The metatarsophalangeal joint is a synovial condyloid type of joint. It plays an important role in flexion and extension of the toes especially during the terminal stance and pre-swing phase of the gait cycle. Isolated metatarsophalangeal joint dislocations are a less common injury [1]. Traumatic dislocation of all the lesser metatarsophalangeal joints in a closed manner is a unique and rare injury [2]. We are reporting a rare case of combined dislocation of all lesser metatarsophalangeal joints.

## Case presentation

**Figure 1:**
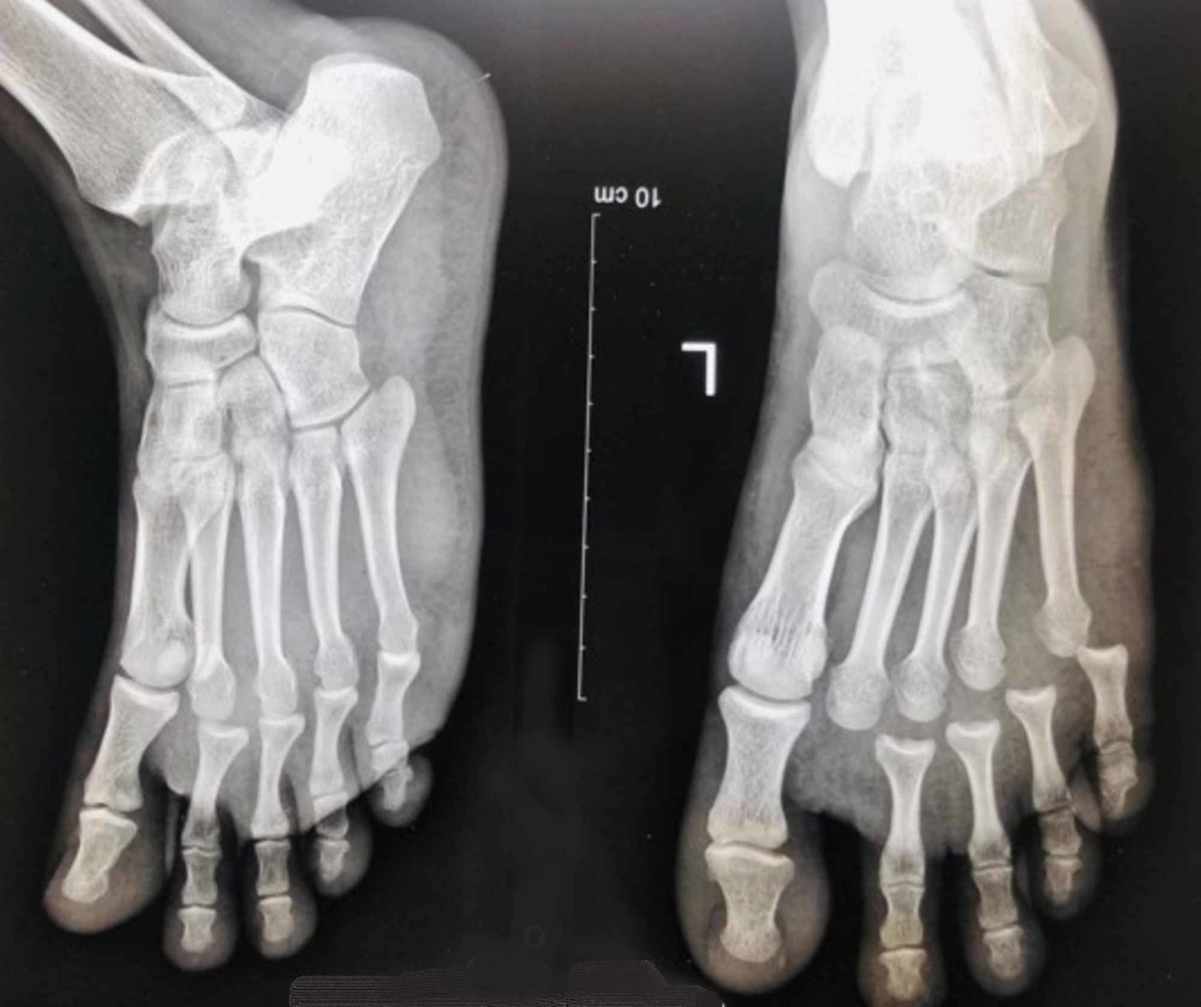
X-Ray of left foot showing dislocations of second to fifth metatarsophalangeal joints with no associated fractures.

**Figure 2:**
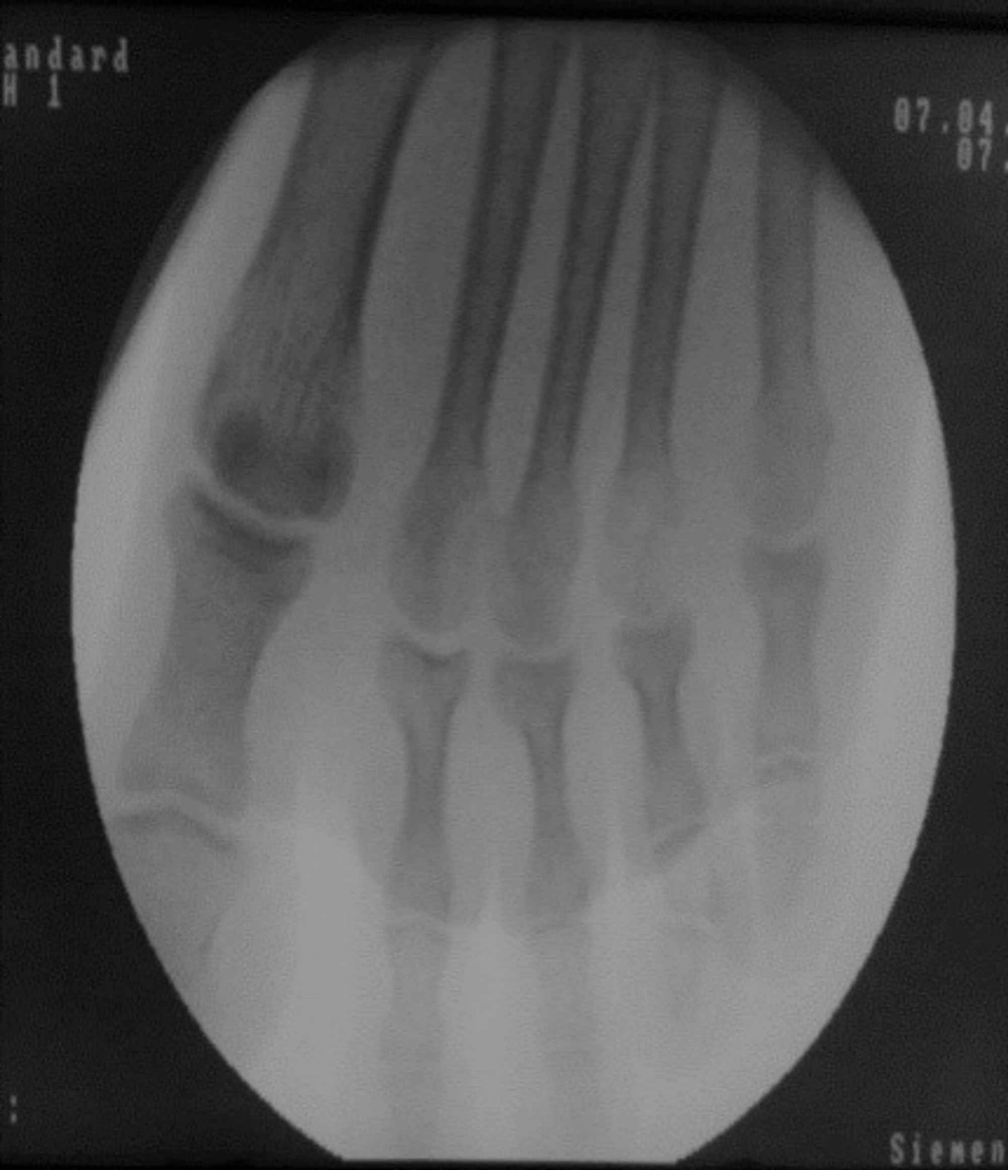
C arm picture showing reduced metatarsophalangeal joints

**Figure 3:**
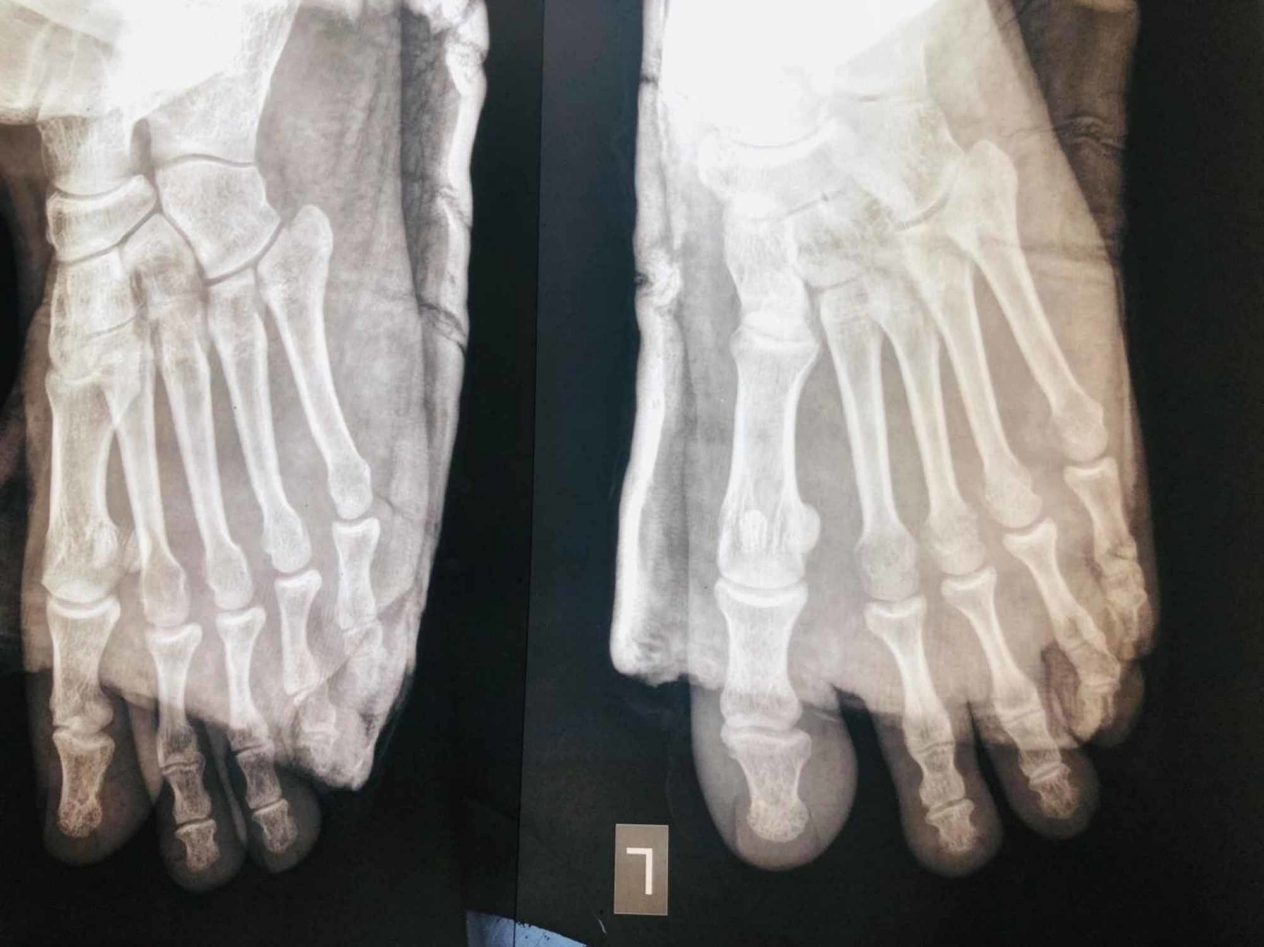
Immediate post operative X-ray shows stable reduction of all metatarsophalangeal joints.

**Figure 4:**
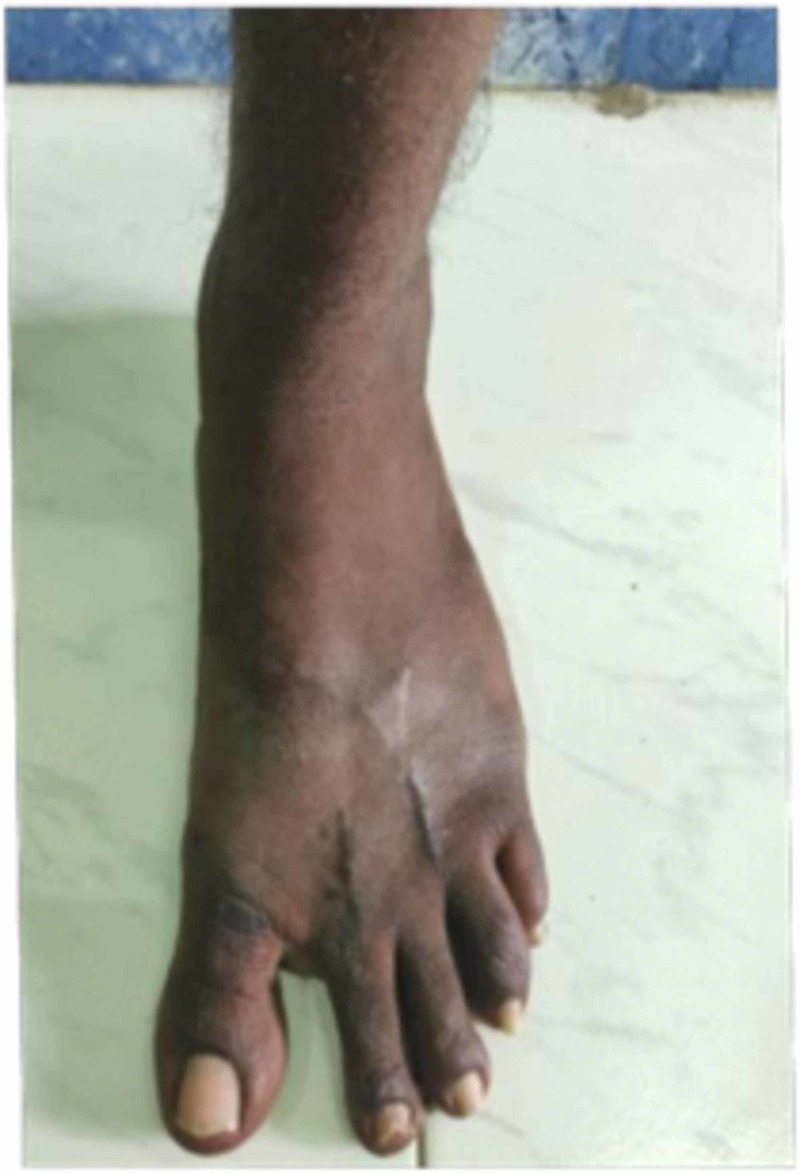
Clinical pics of the patient after he was made to weight bear. He had no deformity or pain during walking.

**Figure 5:**
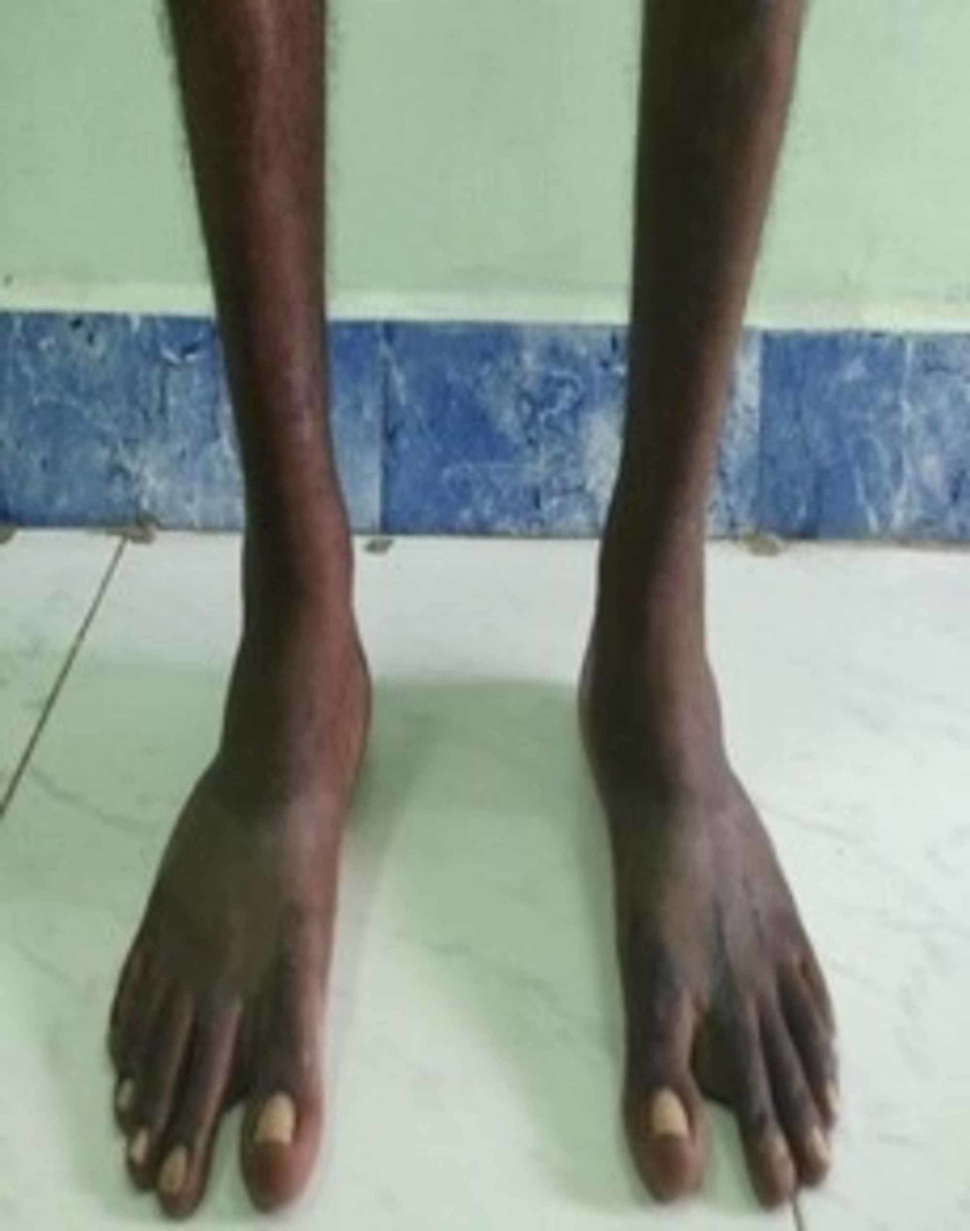
Clinical pics of the patient after he was made to weight bear. He had no deformity or pain during walking.

**Figure 6:**
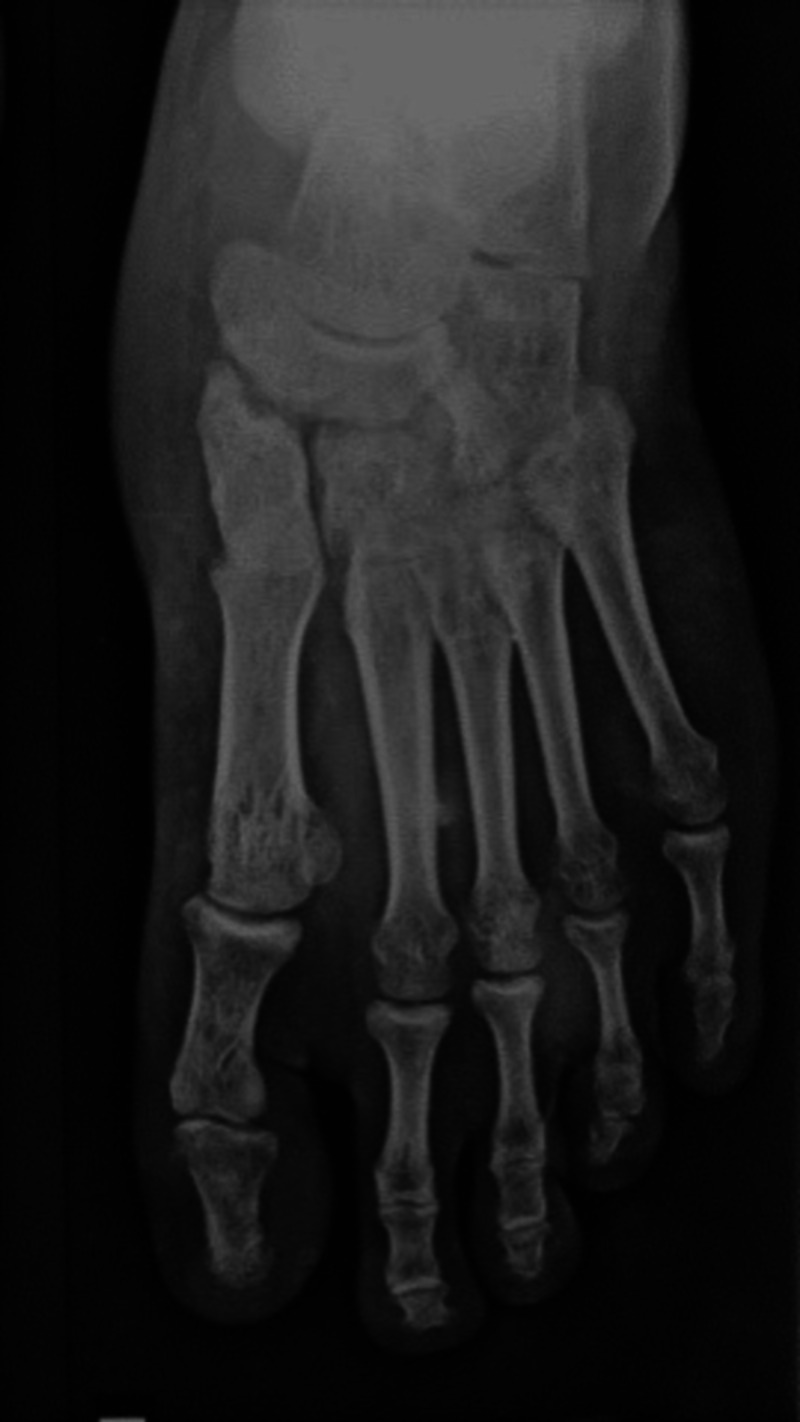
Third month Post operative X rays stable metatarsophalangeal joints

**Figure 7:**
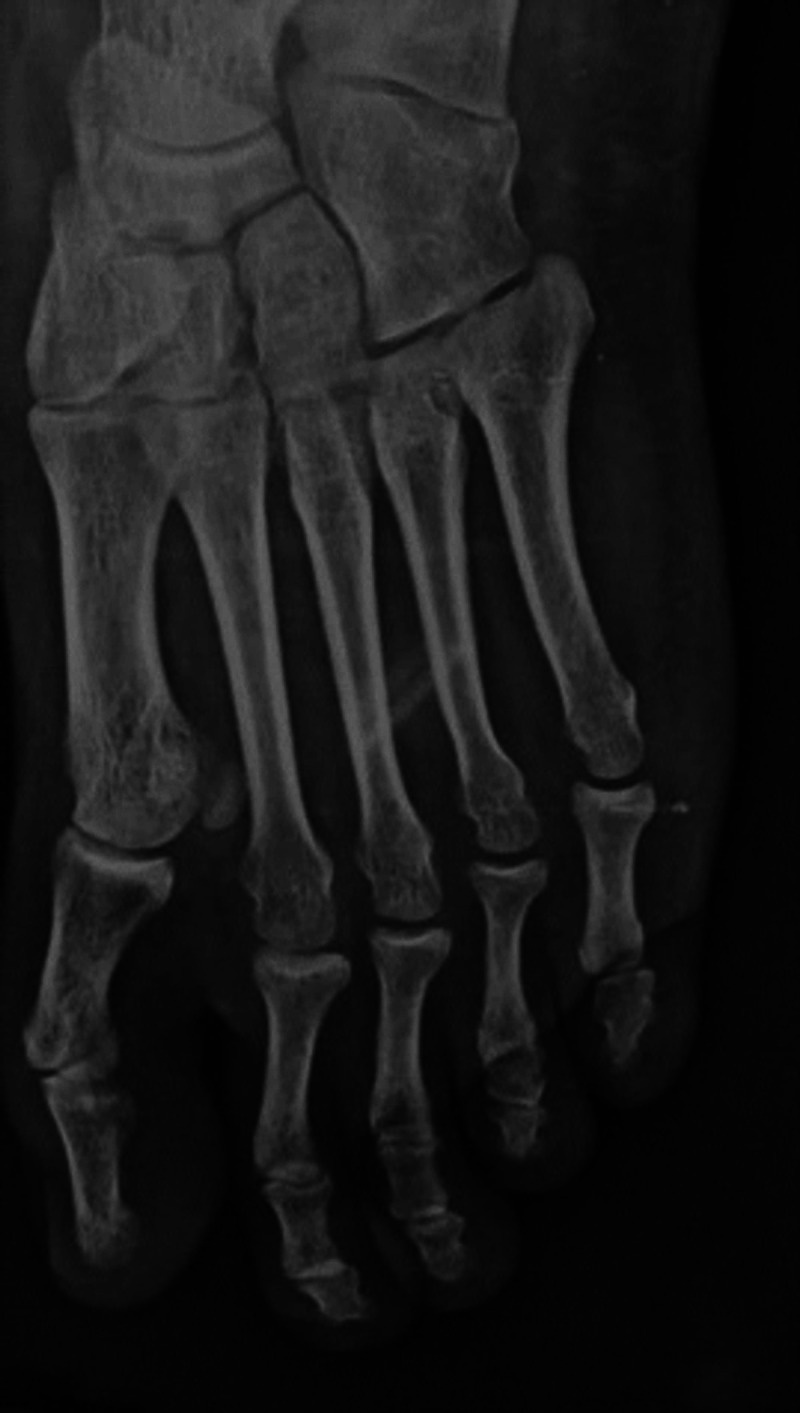
Third month Post operative X rays stable metatarsophalangeal joints

**Figure 8:**
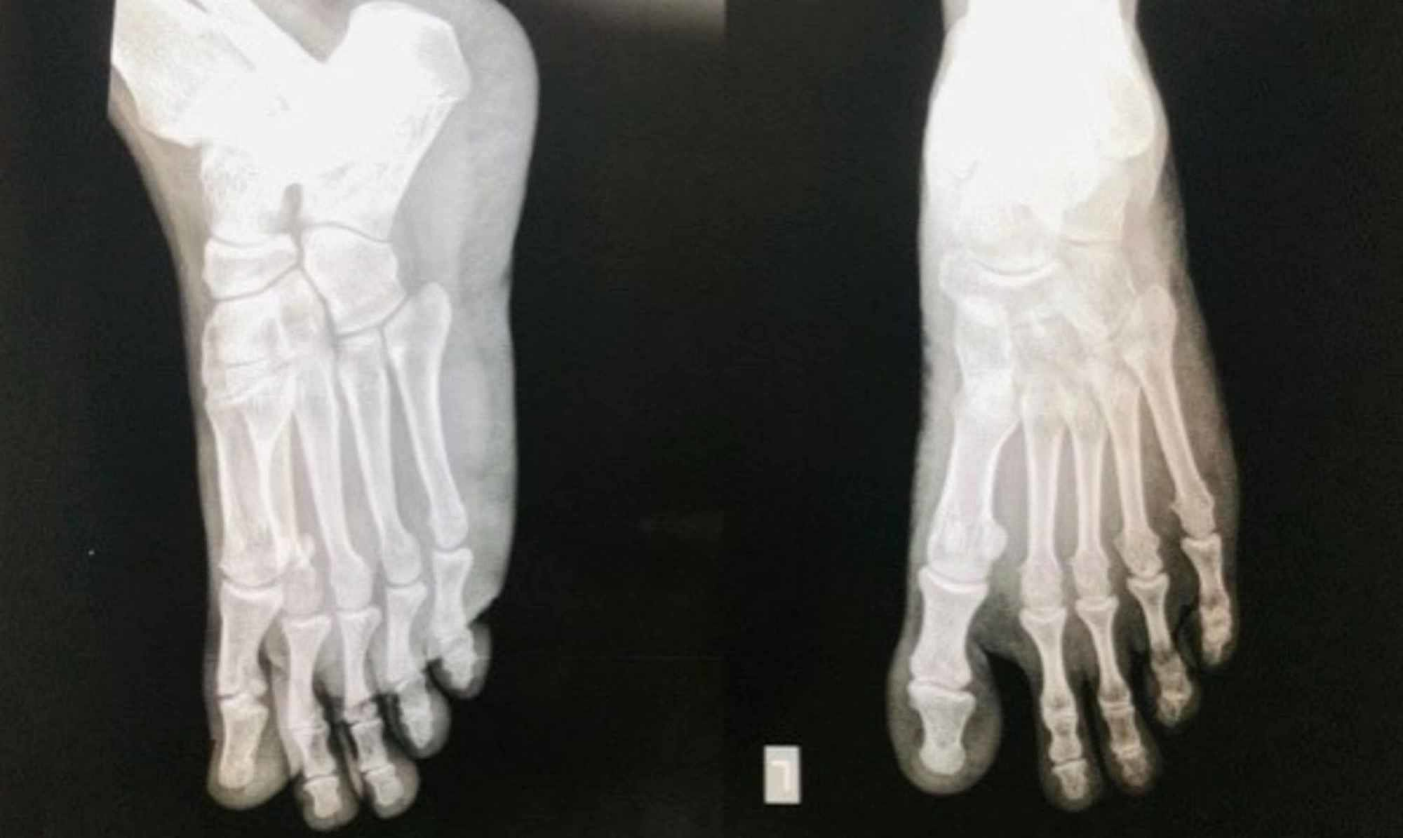
2 years Post operative X rays stable metatarsophalangeal joints

A 34-year-old male patient was brought to the emergency department after a road traffic accident, probably due to force dorsiflexion of the left foot while tiptoeing as the patient tried to gain balance of a stumbling two wheeler and eventually the bike fell on the foot. On examination, swelling and tenderness was noted on the dorsal aspect of left foot. Toe movements of all four lesser toes were painful. Movements of the ankle worsened the pain at the lesser four metatarsophalangeal joints. Plain radiographs of the left foot revealed dorsolateral dislocation of metatarsophalangeal joints of all four lesser toes of the left foot (Figure 1). Patient was taken up for emergency reduction of the joints and under spinal anaesthesia closed reduction was tried with toe traps for all four lesser toes. Three such attempts by different surgeons failed. Under tourniquet control, open reduction of four lesser metatarsal joints was performed using two 3 cm longitudinal incision over second and fourth metatarsal joints respectively. Extensor tendons identified and retracted. Interposition of capsule and longitudinal breach of plantar plate by metatarsal head were noted, which prevented the closed reduction. Reduction of the joint was then performed by incising the plantar plate, capsule was repaired. The steps were then repeated for the other three lesser toes. Congruent joint reduction was then confirmed under C-arm (Figure 2). The joint was stable and reduction maintained without any need for stabilization. Postoperatively a below-knee splint with dorsal re-enforcement in neutral position at metatarsophalangeal joints was given. Dressing was changed on day four and suture removal was done on day 14. Patient was in below-knee slab for four weeks (Figure 3). Progressive weight bearing was started (Figure 4, 5) and patient was reviewed at three and six months and two years (Figure 6-8). Patient was ambulatory without any pain and recurrence.

## Discussion

The metatarsophalangeal joint plays an important role in the formation of the transverse arc of the foot and in plantar flexion immediately before the toe-off stage of walking during the gait cycle. This can only occur if the toes remain in contact with the ground during gait. At the time of pre-swing or toe break stage, the metatarsophalangeal joints dorsi-flex about 90 to 110 degrees. The stability of joints for such a movement is provided by the plantar plate, a dense fibrous thickening of the plantar capsule which is the counter part of the volar plates of the metacarpals of the hand. 

Metatarsophalangeal joint dislocation involving the lesser toes is uncommon. A missed or inappropriately treated multiple metatarsophalangeal joint dislocation will disturb the biomechanics of the foot and gait cycle.

In the literature only three cases of simultaneous dislocation of all the lesser metatarsophalangeal joints have been reported [2-4]. De Palma in 2001 noticed the injury as part of sporting activity and was able to treat the patient conservatively using only closed reduction [2]. Ito et al. documented an accident during an animal attack in 2007 and had to do open reduction to their patients because of difficult reduction [3].

The initial line of management for this injury is an attempt at closed reduction using toe traps. If closed reduction fails open reduction may be necessary. Multiple structures can prevent reduction of the joints namely the volar plate, deep transverse metatarsal ligament, the lumbrical muscles and flexor tendons (short and long). Both dorsal and plantar approaches have been described. In our case we utilized two longitudinal dorsal incisions and retracted the extensor tendons. The plantar plate which obstructed reduction was incised longitudinally and joints reduced gently by rocking movements and traction. Leung et al. in the year 2001 suggested a transverse dorsal incision to avoid problems of wound closure and difficulty in the need for multiple incisions [5]. A plantar incision has the advantage of being able to repair the deeply situated transverse metatarsal ligament and the tense plantar plate. Ideally if the repair of plantar plate is not carried out, then stabilization of the joint using K wire has been suggested [6,7]. In our patient the reduction was stable and hence no internal fixation was used. The order of reduction of the lesser metatarsophalangeal joints has not been clearly described in the literature [1,4,8]. We reduced the second metatarsophalangeal joint first and then sequentially reduced the other three metatarsophalangeal joints.

## Conclusions

Simultaneous dislocation of all the lesser metatarsophalangeal joints is uncommon compared to isolated dislocation of these joints. Closed reduction is often difficult due to interposing of the anatomical structures. In acute presentation, under anaesthesia closed reduction should be attempted, with a simultaneous reduction manoeuvre applied to all the lesser MTP joints. Open reduction should be performed in irreducible metatarsophalangeal joint dislocations to restore the joint congruity and to preserve the plantar plate for better functional outcome.

## References

[1] Bhide PP, Anantharaman C, Mohan G, Raju K (2016). A case of simultaneous traumatic dorsal dislocation of all five metatarsophalangeal joints treated successfully with closed reduction. J Foot Ankle Surg.

[2] De Palma L, Santucci A, Marinelli M (2001). Traumatic dislocation of metatarsophalangeal joints: report of three different cases. Foot Ankle Surg.

[3] Ito MM, Murase KI, Tanaka S, Yamashita T (2007). Dislocation of all metatarsophalangeal joints caused by horse injury. J Trauma.

[4] Neogi DS, Bandekar SM, Sadekar V, Patnaik S, Bhat T, D’Mello Z (2012). Irreducible dislocation of all the lesser metatarsophalangeal joints of the foot: a case report. Foot Ankle Spec.

[5] Leung W, Wong S, Lam J, Ip F, Ko P (2001). Presentation of a missed injury of a metatarsophalangeal joint dislocation in the lesser toes. J Trauma.

[6] Hey HW, Chang G, Hong CC, Kuan WS (2013). Irreducible dislocation of the fourth metatarsophalangeal joint—a case report. Am J Emerg Med.

[7] Kinter CW, Kinter KJ, Hodgkins CW (2019). Surgical repair of an unstable third metatarsopha-langeal joint dislocation via direct plantar plate repair: a simple and effective technique. Int J Foot Ankle.

[8] Dharmshaktu GS, Bhandari SS (2016). Fifth metatarso-phalangeal dislocation with multiple central metatarsal neck fractures in an adolescent: a rare concomitant injury. Int J Orthopaedics.

